# Predicting Hospital Length of Stay at Admission Using Global and Country-Specific Competing Risk Analysis of Structural, Patient, and Nutrition-Related Data from nutritionDay 2007–2015

**DOI:** 10.3390/nu13114111

**Published:** 2021-11-16

**Authors:** Noemi Kiss, Michael Hiesmayr, Isabella Sulz, Peter Bauer, Georg Heinze, Mohamed Mouhieddine, Christian Schuh, Silvia Tarantino, Judit Simon

**Affiliations:** 1 Department of Health Economics, Center for Public Health, Medical University of Vienna, 1090 Vienna, Austria; 2 nutritionDay Worldwide, 1090 Vienna, Austria; michael.hiesmayr@meduniwien.ac.at (M.H.); isabella.sulz@meduniwien.ac.at (I.S.); peter.bauer@meduniwien.ac.at (P.B.); mohamed.mouhieddine@meduniwien.ac.at (M.M.); christian.schuh@meduniwien.ac.at (C.S.); silvia.tarantino@meduniwien.ac.at (S.T.); 3 Division Cardiac, Thoracic, Vascular Anaesthesia and Intensive Care, Medical University of Vienna, 1090 Vienna, Austria; 4 Section for Medical Statistics, Center for Medical Statistics, Informatics, and Intelligent Systems, Medical University of Vienna, 1090 Vienna, Austria; 5 Section for Clinical Biometrics, Center for Medical Statistics, Informatics, and Intelligent Systems, Medical University of Vienna, 1090 Vienna, Austria; georg.heinze@meduniwien.ac.at; 6 Center for Medical Statistics, Informatics, and Intelligent Systems, Institute for Artificial Intelligence, Medical University of Vienna, 1090 Vienna, Austria

**Keywords:** length of stay, nutrition, hospital, survey, discharge, transfer, mortality, dietician, nutrition screening, competing risks

## Abstract

Hospital length of stay (LOS) is an important clinical and economic outcome and knowing its predictors could lead to better planning of resources needed during hospitalization. This analysis sought to identify structure, patient, and nutrition-related predictors of LOS available at the time of admission in the global nutritionDay dataset and to analyze variations by country for countries with *n* > 750. Data from 2006–2015 (*n* = 155,524) was utilized for descriptive and multivariable cause-specific Cox proportional hazards competing-risks analyses of total LOS from admission. Time to event analysis on 90,480 complete cases included: discharged (*n* = 65,509), transferred (*n* = 11,553), or in-hospital death (*n* = 3199). The median LOS was 6 days (25th and 75th percentile: 4–12). There is robust evidence that LOS is predicted by patient characteristics such as age, affected organs, and comorbidities in all three outcomes. Having lost weight in the last three months led to a longer time to discharge (Hazard Ratio (HR) 0.89; 99.9% Confidence Interval (CI) 0.85–0.93), shorter time to transfer (HR 1.40; 99.9% CI 1.24–1.57) or death (HR 2.34; 99.9% CI 1.86–2.94). The impact of having a dietician and screening patients at admission varied by country. Despite country variability in outcomes and LOS, the factors that predict LOS at admission are consistent globally.

## 1. Introduction

Malnutrition and poor eating have been associated with death in the hospital [1]. Identifying patients at risk and providing tailored support has been associated with better outcomes [2]. The PANDORA Score identified six factors in addition to poor eating, which predicted 30-day hospital mortality [3]. Nutrition care is a fundamental aspect of a patient’s hospital experience but little attention has been paid to optimizing nutrition care resources in the hospital setting when compared to optimizing medical or nursing care. Measuring the effect of nutrition care on patients’ clinical course poses a challenge for stakeholders trying to make evidence-based decisions about the employment of dieticians, establishment of nutrition teams, and the adoption of screening tools and individualized use of nutrition therapies. Particularly lacking is information on the structural factors of nutrition care, such as dietician staffing and nutrition care processes, as well as impacts of weight loss prior to hospitalization. This information is important because it can trigger further clinical management or add to the measurement of clinical parameters, such as body mass index (BMI), fat free body mass (FFM), inflammatory markers, and waist circumference. Clinical parameters in the hospital take time and also depend on resources, thus if information from the patient at admission could be used as a precursor or proxy to clinical parameters, it could potentially lead to more efficient care.

Length of stay (LOS) is often used to measure the effectiveness of hospital care because it may represent a better clinical course and improved quality of life, and because it is associated with healthcare costs. In many countries, hospitals are the most expensive care setting [4]. LOS is a key indicator of inpatient resource use and hospital efficiency around the world and is relevant as both a clinical outcome and an economic outcome [5,6]. Factors that influence LOS may differ by country, indicative of cultural and health care practices. The gate keeping function of primary care influences the case mix of patients admitted to hospitals. The availability of post-hospital care, including rehabilitation facilities, ambulatory nursing services and hospice, also impacts the role of the hospital [4,5,6]. Financial incentives from insurance systems, such as DRG based reimbursement, could incentivize discharging patients after a certain point in time [4]. Cultural expectations of location of care determine to what extent the patient recovery takes place in the hospital versus at home and vary between countries [5,6,7,8,9]. Despite these differences, the pursuit of ways to reduce hospital LOS appears to be a priority across countries [5,10,11,12].

Identifying drivers of LOS may promote cost containment, improve efficiency, and improve patient outcomes [7]. Many studies focus on a specific high cost disease area [13,14], procedure [15,16,17] or hospital specialty, such as intensive care units (ICU) [18,19]. There is a lack of overarching evidence on what determines LOS in the hospital in general, including all departments and services. Evidence of this sort would allow for the promotion of efficiency across the board rather than in individual hospital departments. OECD data, which provide a global overview of LOS in hospitals does not take into consideration the case mix, patient characteristics, and hospital care structure. This study aims to identify the predictors of LOS, which are present at the time of admission to the hospital, making use of an international prospective uniformly collected database with a specific focus on the role of nutrition-related variables. Furthermore, it aims to describe the variations by world regions and countries.

## 2. Materials and Methods

### 2.1. Study Design

This study is based on annual repeated cross-sectional global observational data (*n* = 155,524) from a voluntary international ESPEN funded audit called “nutritionDay”, which began in 2006 with the aim of collecting data on hospital nutrition care (www.nutritionday.org) [20]. In four parts, the questionnaire collects information about the hospital department, about the patient from the medical professional’s perspective, and about the patient from the patient’s perspective is collected on one day a year from all consenting inpatients in a participating hospital department and is followed up 30-days later with an outcome collection (discharged home, transferred to another facility, or died in hospital). A detailed description of the nutritionDay survey has been previously published [21]. The data are anonymized. The collection of the data was approved by the Ethical Commission of the Medical University of Vienna (EK407/2005).

### 2.2. Study Population

The nutritionDay data population from 2006–2015 contains 155,524 patients admitted to 8336 departments in 3177 hospitals in 60 countries. Although nutritionDay data are available up to 2020, the utilized data are up to 2015 as the survey questionnaires were changed in 2016. Starting in 2016, two new versions (one short version and one long version) of the survey replaced the versions that was being used for 10 years. The new versions have different questions focusing on quality of care and economic processes, and thus no longer only have the original aim of understanding nutrition care processes in general.

### 2.3. Variables

The outcome of LOS can occur in three ways, or “event types”: discharged home, transferred, or in-hospital mortality. LOS was defined as the time from admission until the time to discharge, transfer, or in-hospital mortality. Patients who were still in hospital at the 30-day follow-up were censored. Variables of interest were selected for inclusion before any analysis took place, based on whether they were factors that were present at hospital admission and considered to be clinically relevant by the study team. Variables from two hierarchical levels were considered: those that referred to the care structure and those that defined patient characteristics. Structure characteristics used were department specialty, department bed occupancy, dietician available to the department, dedicated nutrition care person at the department, nutrition team at the hospital, and nutrition screening at admission. Nutrition-related variables were patient and nutrition care structure variables considered relevant for the provision of nutrition care in the hospital. At admission, there was one nutrition-related patient characteristic: weight change in the last three months. There were four nutrition-related structure characteristics: nutrition team, nutrition care person, dietician available, and screening at admission (further variable descriptions provided in Appendix A.1).

### 2.4. Statistical Methods

Statistics were carried out using R 3.6.1 (R Development Core Team 2019) [22], and the survival package [23,24]. The statistical analysis consisted of a descriptive and multivariable part. The descriptive analysis looked at median length of stay per variable and per country, as well as cumulative incidence estimation of length of stay. The multivariable analysis looked at the global data as well as per country analysis.

#### 2.4.1. Descriptive Analysis

The descriptive statistical analysis consisted of baseline characteristics of the included patients, LOS calculations with length bias adjustment [25] for each variable, the distribution of outcomes per country, and the estimation of cumulative incidence of time to the three event times by the Aalen–Johansen estimator adjusted for length bias [26,27].

#### 2.4.2. Multivariable Analysis Statistical Methods

The effects of care structure, patient, and nutrition-related variables on the cumulative incidence of discharged, transferred, and in-hospital mortality were then investigated using a multivariable Cox proportional hazards (CPH) model for cause-specific hazards accounting for competing risks [28]. The selection of variables for inclusion were based on three criteria: (1) available at the time of admission, (2) clinically relevant, and (3) not missing in more than 50% of patients. The reference categories were selected via clinical expertise of project leader or by using the category or value containing the median of the underlying continuous distribution. Thus, the reference for age was the category “61–70 years old”, for bed capacity was “low to middle capacity”, for dietician was “none available”, for specialty was “internal medicine”, for weight change in the last three months was “idem”, for regions was Europe Region A (defined in Table S1), for screening of patients was “yes”, for year was “year 1”. Data from 2006 were not included because the variable about screening had not yet been included in the questionnaire. The reference year was thus 2007. All other variables were dichotomous, including affected organs and comorbidities. The marginal R2 method was used to test each variable’s impact on the explanatory power of the multivariable model [29]. For the global multivariable model only, a more stringent statistical significance cutoff of 0.001 was used to describe effects, along with effect sizes and confidence limits due to the large sample size [30].

CPH regression for time-to-event data was applied to LOS to model cause-specific hazards accounting for competing risks, clustering by hospital department and correction for length bias by appropriate weighting. The robust sandwich covariance was used to compute confidence intervals for estimated hazard ratios [31]. For care structure characteristics, this covariance was evaluated at the hospital level. Three types of events were considered: discharged home, transferred, and died in hospital. To assess the performance of the models, discrimination via the incident/dynamic C-statistic which accounts for left-censoring of data was derived [32,33]. The proportional hazards assumption was checked using the Schoenfeld residuals test of independence between time and residuals for each variable [33,34]. Statistically significant nutrition-related variables were examined individually by multiplying them by time to ensure that there was no indication of a departure from the proportional hazards assumption. Baseline hazard was examined graphically to confirm that hazards over time were consistent with expected clinical course.

#### 2.4.3. Country-Specific Analyses

Exploratory country analysis was conducted by applying the multivariable CPH model in each country with a complete case sample size above 750 to shed light on country-level differences in predictors of LOS. Countries with a complete case sample size of above 750 were considered for the country-specific sensitivity analysis on the predictors of LOS with a focus on nutrition-related variables in the reporting (the results per country are included in Tables S2–S10 in the Supplementary Materials). In the country-specific analysis, the same variables were used as in the global model, except “region” was taken out due to irrelevance, “year” was taken out due to different cycles of participation in each country, gained weight and idem were combined for weight loss in the last three months, and the number of levels in the variable “specialty” were merged into broader categories. The combined specialties were internal medicine, surgery, geriatrics, neurology, ear-nose-throat (ENT), gynecology, and others. Where a country had no values for a variable, the variable was excluded to avoid nonconvergence. In the country models for time to death in hospital and time to transfer, the affected organs were reduced to lung, liver, skeleton/bone/muscle, cancer, and infection. Department specialty was excluded. In some cases, the number of events for the outcomes death or transferred was too small leading to lack of convergence of the models. Thus, for each country, only the results of the outcomes for which the model converged are reported. Variables in country analysis were considered statistically significant at a level of *p* < 0.05, less stringent than the *p* < 0.001 for the global model, due to the smaller sample sizes for the country models.

#### 2.4.4. Cross-Sectional Study Bias

LOS bias correction was applied to improve the generalizability of the study according to methods designed and tested on nutritionDay data to correct for cross-sectional sampling bias that leads to patients with longer LOS being overrepresented [25].

#### 2.4.5. Department Clustering

All analyses used department clustering to adjust for the assumption that patients are more similar in a single department, and to avoid overrepresentation of larger units in the effect size of structure variables that were collected on a department level [28].

### 2.5. Missingness

Most missing values were in “structure” characteristics, which were missing due to the entire “department” section of the questionnaire not being filled out by the respective department. This information could not be predicted from patient characteristics, thus no imputation was considered. Additional categories coding the missingness of a value were created for variables for which missingness could be clinically meaningful (mentioned above). All analyses were based on complete cases, and sample sizes per country are provided accordingly.

## 3. Results

**Figure 1:**
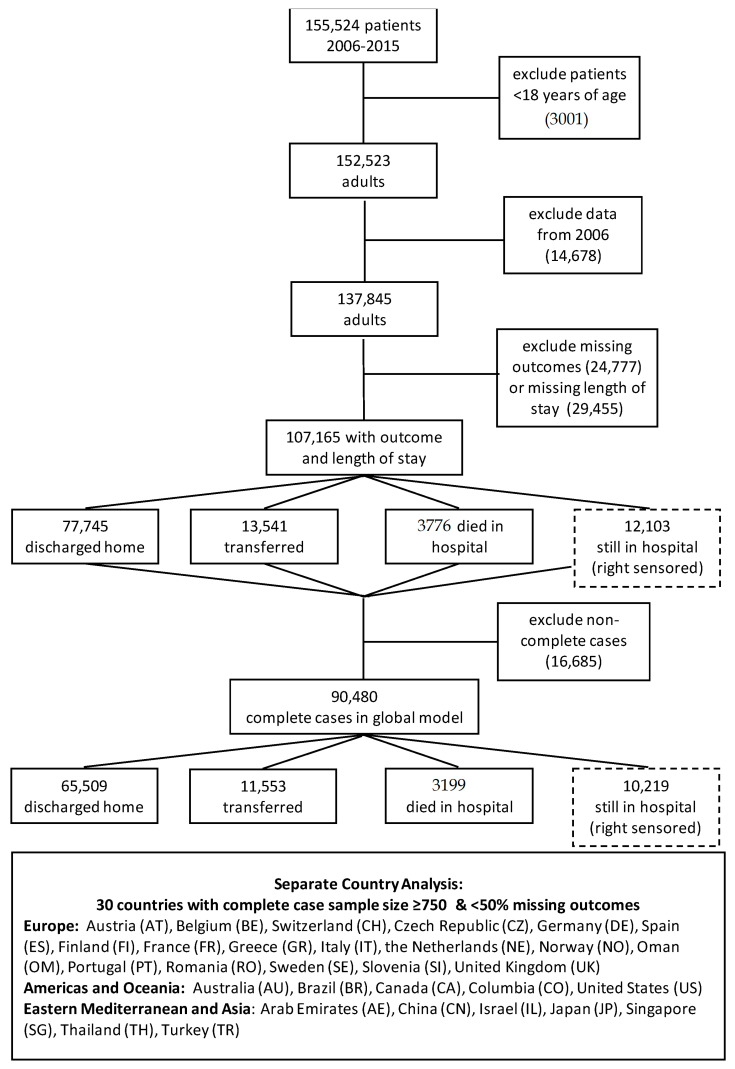
Participant Flow.

A total of 90,480 complete cases were used for the global analysis (Figure 1). The eligible population of complete case adults did not differ significantly in baseline characteristics from the original population of 155,524 (Table S11 and Appendix A.1).

### 3.1. Outcome Timing

**Figure 2:**
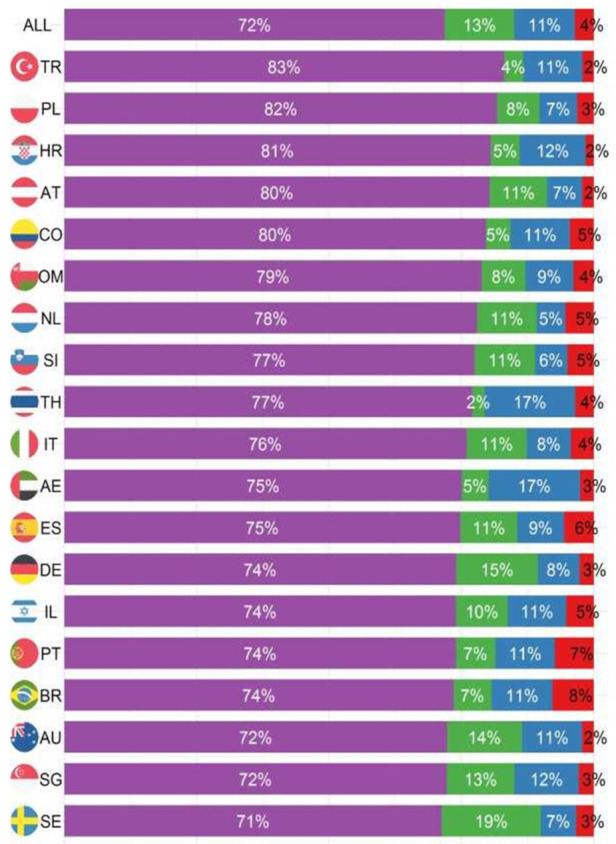

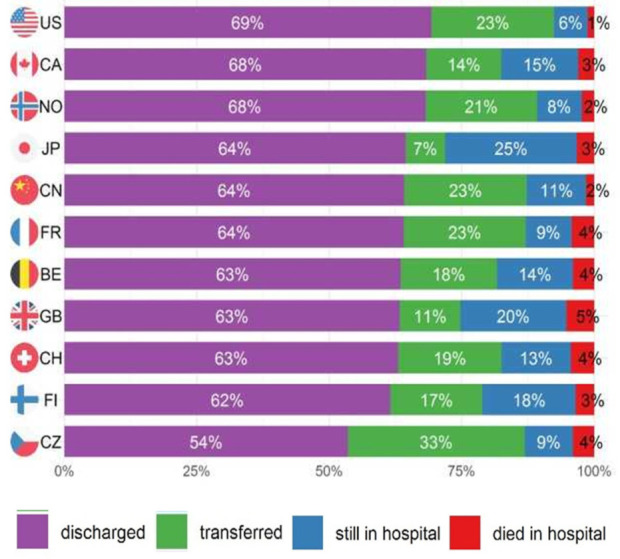
Proportion of outcomes 30 days after nutritionDay by country.

**Figure 3:**
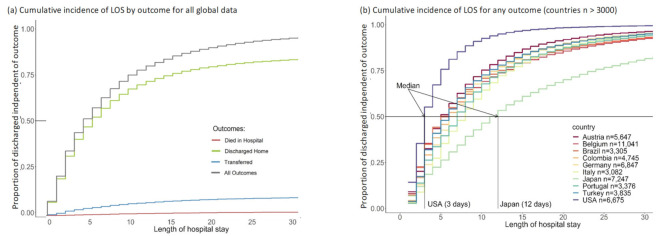

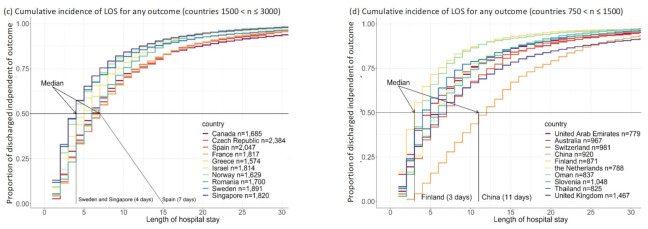
Cumulative incidence graphs for global data and per groups of 10 countries (adjusted for length bias) including figure (**a**) for the global data and figure (**b**) for countries with *n* > 3000; figure (**c**) for countries with 1500 < *n* ≤3000; figure (**d**) for countries with 750 < *n* ≤ 1500.

Cumulative outcomes were primarily driven by the event “discharged”, since this is the event with the highest proportion in each country (Figure 2). The cumulative incidence of the three event types differed considerably, with most people having been discharged home (Figure 3a). The decision to transfer appears to be later than the decision to be discharged (Figure 3a). The cumulative incidence function for each country adjusted for cross-sectional length bias are described in Figure 3b–d. For example, patients in China tend to be kept in the hospital for at least three days before any events occur, and then are discharged gradually, at a median of 11 days. The country lines also level off at different points, meaning by a certain number of days, nearly all events have occurred. In most countries, this appears to be at 25 days.

The European countries are in the same pattern of event timing, with the exception of Finland (Figure 3d). Finland appears to have a pattern similar to the US with most events occurring in the first three days and a levelling off at about 6–10 days with a flat tail thereafter. Oman also has a distinctive pattern, with a median similar to the core European countries at four days, but most patients discharged by 10 days, more similar to Finland and the US.

### 3.2. Length of Stay (LOS)

The median LOS adjusted for length bias is 6 days (25th percentile 4; 75th percentile 12) in the global population (*n* = 90,480). Changes in median length bias adjusted LOS can be observed by age, specialty, region, weight loss, affected organs, and in comorbidities such as diabetes (Table 1 for all nutrition-related variables and Table S1 for all variables). These differences in the nutrition variables, for example a median LOS of 7 (4–12) for those not screened and 5 (3–10) for those who are screened, show that in isolation, these variables seem to impact LOS. The multivariable analysis will examine these variables adjusting for the effects of all other included variables to ascertain whether they are indeed predictors of length of stay.

**Figure 4:**
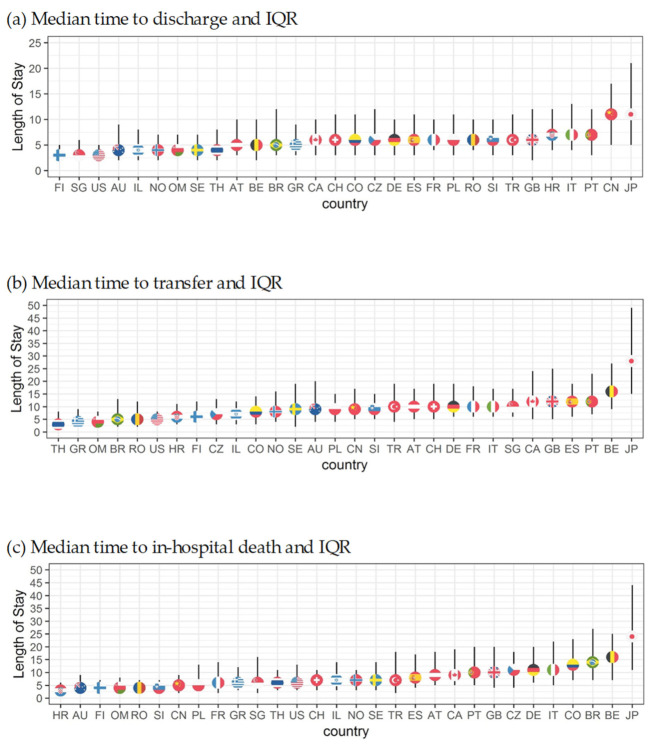
Median length of stay for all outcomes by country corrected for length bias.

How long patients stay hospitalized until they experience one of the three event types is shown with median LOS and 25th to 75th percentile (Figure 4a–c). Finland (3; 2–5), Singapore (3; 2–7), and the US (3; 2–5) had the shortest times to discharge, and China (11; 5–17) and Japan (11; 5–21) had the longest. Thailand (3, 3–8) and United Arab Emirates (3; 3–10) had the shortest time to transfer while Japan (28; 16–49) and Belgium (16; 9–27) had the longest. Time to in-hospital mortality was the shortest in Romania (4; 3–7), Oman (4; 2–10), and Australia (4; 2–9) and longest in Japan (27; 9–49) and Belgium (16; 7–25). This indicates differences depending on country, which may be structural or cultural.

### 3.3. Multivariable Analysis

Patient characteristics dominated over hospital care structure characteristics in predicting LOS from admission in the multivariable analysis (Table 2 with all significant variables only, and Figure S1 with all variables). The global model C-statistic was 0.59 for time to discharge, 0.53 for time to transfer, and 0.55 for time to death.

#### 3.3.1. Predictors of LOS

Increasing age was associated with increasing LOS for patients until discharged, shorter time to in-hospital mortality, and faster time to transfer (Table 2 for statistically significant variables and Figure S1 for all variables). Sex was not associated with time to discharge or transfer, but being male was associated with shorter time to in-hospital mortality. Of the 16 affected organs, “brain and nerves” and “skeleton and bone and muscle” and “infection” were significantly associated with the hazard for three event types. Having “brain and nerves” as an affected organ increased predicted time to discharge, decreased predicted time to transfer, and decreased predicted time to in-hospital mortality. Having “skeleton and bones and muscle” as an affected organ, was associated with an increased time to discharge, decreased time to transfer and increased time to in-hospital mortality. Having an infection increased predicted time to discharge, decreased predicted time to transfer, and decreased predicted time to in-hospital death. For other organs, only some outcomes were significantly associated with LOS (Figure S1). Nutrition-related structural variables (Table 2) that predicted LOS were “dietician available”, which was significantly associated with increased length of stay for transferred patients and “nutrition team available” which was significantly associated with increased length of stay for discharged patients. Weight loss and not knowing if one had lost weight and missing information in this category remained important in the multivariable analysis, showing an increase in length of stay for patients discharged, and a decrease for those dying in hospital.

#### 3.3.2. Country-Specific Analyses

**Figure 5:**
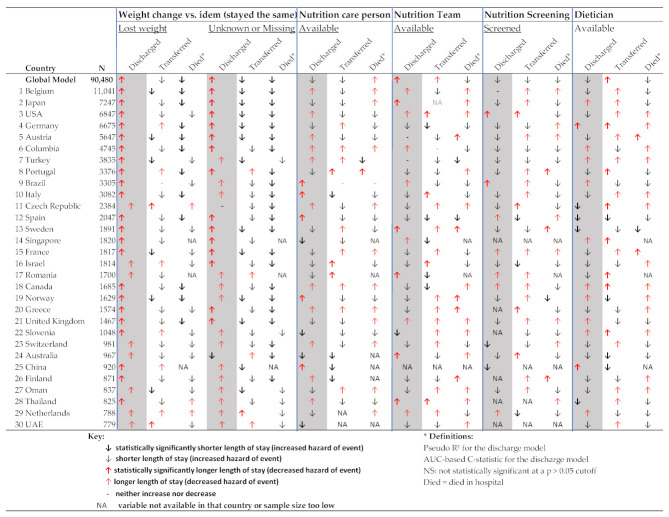
Nutrition-related determinants of length of stay (based on multivariable Cox proportional hazards competing risk country models).

The country analyses show the importance of patient characteristics, such as age in determining LOS (Tables S3–S11). In Figure 5, the nutrition-related variables are shown individually for all countries and the global model. It shows that selecting “having lost weight in the last three months” was associated with statistically significantly longer time to discharge in 23 out of 30 included countries and not knowing whether one has lost weight or missingness of that variable was associated with a longer time to discharge in 13 out of 30 included countries. Meanwhile, the role of nutrition characteristics (nutrition care person, nutrition team, nutrition screening, and dietician) varied from country to country; sometimes these variables were associated with an increase and sometimes they were associated with a decrease in predicted LOS, explaining some of the variability in the global model. The model fit statistics improved in many of the country-specific analyses compared to the global model, suggesting that the role of variables may differ between countries.

## 4. Discussion

### 4.1. Discussion

This is the first study to present median LOS and its context in different countries and to explore LOS predictors present at patient admission of LOS leading to discharge home, transfer to another facility, or death in hospital. Consistent globally, having lost weight, or reporting that weight loss was unknown in the last three months prior to hospitalization was associated with longer LOS for those discharged from the hospital, and shorter LOS in those who were transferred or died in the hospital. This was irrespective of country, age, or the patients’ affected organ(s). To date, no other studies have assessed the impact of weight loss prior to admission on LOS or the impact of asking about previous weight loss when the patient is admitted to the hospital as a potential screening tool to identify patients who need an additional nutrition assessment or nutrition care. Weight loss is a simple patient-reported nutrition-related factor, which is easy to collect information on compared to formal clinical parameters that need proper measurement. Our study is indicative that weight loss prior to admission is a good proxy indicator for more complex clinical measurements, such as inflammatory markers, FFM muscle mass and function measurement, BMI, waist circumference, and monitoring eating over several days. Indeed, some studies have shown that receiving early, tailored nutrition care may lead to better outcomes and be more cost-effective [2]. Collecting this information as part of routine care may help direct the patient toward better nutrition care in cases in which the weight loss is unintended and modifiable with nutrition treatment.

Nutrition-related structure factors at admission, such as having a dietician available in the department, were sometimes associated with a longer LOS, and sometimes with a shorter LOS, depending on country. The varied direction of the effect of nutrition care variables could represent the effect of nutrition care on LOS, or they could act as a proxy for a size of the hospital, level of resources, and processes in place. An increase in predicted LOS due to these variables could represent better care, more time in care, more severe underlying disease, or worse outcome. A decrease in LOS could represent worse care, less time in care, less severe underlying disease, or better outcome. In some countries, screening at admission has been shown to be related to an increase (Greece, Switzerland, China, Oman) or a decrease in LOS (United States, Germany). It could be that screening is conducted only for patients who are expected to stay longer, or that screening is only linked with processes that reduce LOS in some countries. The lack of predictive effects in other countries may be explained by the fact that screening itself is not enough, and the actions that follow screening are more important. These actions, such as providing oral nutritional supplements, may only be relevant for a small proportion of patients. Such an effect could be better teased out in studies that look at LOS in patients who have been screened compared to those who have not and also follow up on their treatment as a result of the screening. Furthermore, it may be a small subsample of patients requiring additional nutritional care, which may not be large enough to show an effect in the general hospitalized population. This study also suggests that asking the patient whether they have lost weight in the last three months on an admission form may be a simple way to screen them in a busy, general hospital setting where there are limited staff resources to conduct more thorough screening.

LOS is a function of the role of the hospital in the healthcare system, cultural expectations, and patient and structure characteristics. The role of the hospital depends on the reimbursement system, which incentivizes certain types of care in certain healthcare settings and on the institutions available to the patient before, during, and after hospitalization. Some of these resources include the gate-keeping of a strong primary care structure, hospice services for patients who are terminally ill, rehabilitation services for patients still in recovery, and even the number of hospitals in a catchment area leading to more or fewer available beds. Cultural factors, such as preferring death at home to death in hospital, preferring to recover fully at home under the care of family members instead of at the hospital, and preferences about pain management at a hospice are also relevant in determining LOS. Our analysis indicates that discharge, transfer, and death have different incidence and time profiles in different countries. Cumulative incidence visualizes country-specific profiles. Countries, such as US and Finland, with a well-diversified choice of settings, and rigidly structured healthcare system that promotes utilization of secondary care are characterized by a short LOS and high proportion of patients transferred. In such countries, it is unlikely that the characteristics of patients themselves or the structure characteristics of an individual hospital may impact LOS. Conversely, countries with a longer LOS and low proportion of patients transferred such as Japan, the hospital plays a more central role in medical care for a diverse case-mix of patients, and patients are not released until full recovery. In such a system, it may be more likely that factors such as nutrition-related structure or patient characteristics could impact LOS if they contribute to recovery of the patient. There was no systematic tendency for association of nutrition related factors with LOS besides weight loss in the last three months prior to admission. This does not exclude that nutrition related factors may have a differential impact on LOS in different countries or regions, or in specific hospital departments.

### 4.2. Country Comparisons

This is not the first comparative study on LOS with a global reach. The OECD Health Statistics compares hospital average length of stay (ALOS) between countries [4]. ALOS is used for monitoring and analysis to evaluate healthcare systems. The nutritionDay study uses median LOS to understand the pattern of LOS in the typical hospital inpatient setting and identify what influences it on the hospital level. The OECD data are reported on a country level rather than a patient level; therefore, they cannot be used to discover predictors of LOS in such detail. nutritionDay data are consistent in its definition of LOS, whereas in the OECD data, countries measure and report LOS differently. Despite these differences, similar trends can be observed. As reported by the OECD, in 2015, Japan had the highest ALOS of 16.5 days for curative care and 29 days for all inpatient care, with the next highest country being Korea with 16.1 days. The lowest ALOS in the OECD data was for Turkey at 3.9 days. The US was the 8th lowest out of 40 countries represented in the data with an ALOS of 6.1 days. However, there were some marked differences. For example, Finland had one of the highest ALOS at 9.4 days according to the OECD, while having a median LOS of 3 in the nutritionDay dataset, which made it one of the countries with the lowest median LOS. This discrepancy may indicate that although most patients have a lower LOS in Finland, high tech services available in the hospital may lead to a small percentage of patients who stay for a very long time and thus contribute to a higher average LOS.

### 4.3. Limitations, Mitigations, and Strengths

A major limitation of this study is in the selection bias resulting from the voluntary nature of the survey; participating departments tend to be more interested in nutrition care, may have implemented more nutrition care measures, and thus potentially have different outcomes from those who did not participate. The fact that it is not known if the participating hospitals were private or public, urban or rural, or have other characteristics that distinguish this group from the average hospital in the country further limits the generalizability of the country analyses. Nevertheless, our study has the largest prospective sample that applied a similar methodology in all countries.

The cross-sectional study design is another limitation of this study, which results in an over-representation of those patients who were in the hospital longer and does not allow for a more exhaustive collection of variables at admission that may be associated with LOS. By design, the survey does not collect physiological or biochemical markers at admission for which, if it had been collected, may have reduced unexplained variability in the model. The overall discriminatory power of the model was low, but some predictors still seemed to be more relevant than others as exhibited by the significance tests of their regression coefficients in the multivariable models. The purpose of this model was to identify the direction and magnitude of the association for variables at admission, and for this, the C-statistics show that the results from this analysis are certainly beyond chance, irrespective of the variability introduced by the wide mix of countries. To understand the complexities that influence entire LOS, an understanding of what happens during the hospital stay is also necessary [35]. Another limitation is the large fraction of missing data. This issue is most likely to impact the country-specific results, as the baseline characteristics in the complete cases global dataset were not different from the full global dataset (Table S11). Finally, the study only reveals possible predictors of LOS, but cannot show a causal relationship with LOS, such that altering variables would cause a decrease in LOS.

Despite the above limitations, this is the first study to compare the association of structure and patient characteristics at admission with LOS in different countries, with the assumption that different patient and structural factors may be associated with LOS differently or have a different magnitude of effect depending on the health system structure. It is also the first study to examine LOS and nutrition-related variables on a general hospital population, rather than in a specific disease area or department type. Additionally, nutritionDay data are well suited for such an analysis with its large sample size, multi-national participation, and focus on nutrition care factors, while not neglecting the collection of important known determinants of LOS, such as comorbidities. The evidence from the multivariable analysis showing expected results, such as an increased LOS for those with infection, older patients, stroke, and in the geriatrics department are an indication of the appropriateness of the nutritionDay data and credibility of the study results. In previous studies, nutritionDay data were used to show how nutrition related factors during hospitalization predict in-hospital mortality [1,36,37,38] and a simple predictive score for 30-day in hospital mortality was developed called the PANDORA score [3].

## 5. Conclusions

Cross-sectional data allows an estimation of country-specific LOS adjusted for patient characteristics and for affected organs as well as the consistent methodology of data collection makes it possible to compare nutrition parameters present at admission within the context of health care systems across countries. At admission, patient characteristics, such as age and affected organs and the country of hospitalization, were the most robust predictors of LOS. Additionally, the self-reported nutrition parameter of weight loss within the last three months was also associated with significantly longer time until discharge in the multivariable global model and in the country-specific multivariable analysis. Country-specific median LOS varied by a factor of four in patterns similar to published OECD data. Using simple parameters such as “weight loss in the last three months” as screening tools at admission may help the provision of more targeted nutrition care and more efficient identification of patients needing more timely measurement of additional nutrition-related clinical parameters.

## Figures and Tables

**Table 1:** Median length of stay by selected * nutrition-related baseline variables adjusted for length bias.

	Total *n* = 90,480	Time to Discharge *n* = 65,509	Time to Transfer *n* = 11,553	Time to Death *n* = 3199
Weight Δ in the last 3 months (prior to hospitalization)				
Lost weight	7 (3–13)	6 (3–12)	10 (4–19)	14 (7–26)
Idem (stayed the same)	4 (3–9)	4 (2–8)	7 (3–14)	11 (5–21)
Gained weight	4 (3–8)	4 (2–8)	7 (3–13)	9 (3–22)
Unsure	7 (3–13)	6 (3–12)	10 (5–18)	12 (5–21)
Missing	6 (4–12)	4 (2–9)	10 (5–20)	10 (6–20)
Nutrition risk screening at admission				
Not Screened	7 (4–12)	5 (3–10)	10 (5–17)	12 (7–22)
Screened	5 (3–10)	5 (3–9)	8 (4–16)	12 (6–24)
Missing	5 (4–11)	5 (4–11)	3 (2–6)	2 (2–2)
Dedicated nutrition care person (department)				
Yes	5 (3–10)	5 (3–9)	8 (4–16)	13 (6–24)
No	7 (4–13)	6 (3–11)	9 (5–18)	11 (5–22)
Nutrition team available (hospital)				
Yes	6 (4–12)	5 (3–10)	10 (5–18)	13 (7–24)
No	5 (3–9)	5 (2–9)	7 (3–14)	11 (5–20)
Dietician available				
Yes	5 (3–10)	5 (3–9)	8 (3–15)	12 (6–23)
No	7 (4–12)	5 (3–10)	10 (5–18)	13 (6–24)
Missing	6 (3–11)	5 (3–10)	10 (5–19)	12 (6–22)

* Median length of stay for all baseline variables are available in the Supplementary Materials; outcomes are measured 30 days after nutritionDay.

**Table 2:** Hazard ratios and confidence intervals for statistically significant * determinants of length of stay (based on multivariable global model **).

	Discharged		Transferred		Died in Hospital	
Variable	Increase LOS	Decrease LOS	Increase LOS	Decrease LOS	Increase LOS	Decrease LOS
Patient characteristics						
Age (reference 61–70)	-	-	-	-	-	-
18–30	-	1.18 (1.12–1.24)	-	-	0.31 (0.14–0.71)	-
31–40	-	1.15 (1.09–1.21)	-	-	0.49 (0.29–0.85)	-
41–50	-	1.10 (1.05–1.15)	-	-	-	-
51–60	-	1.06 (1.02–1.10)	-	-	-	-
71–80	0.92 (0.89–0.96)	-	-	1.25 (1.10–1.43)	-	1.40 (1.10–1.77)
81–120	0.78 (0.74–0.82)	-	-	1.77 (1.54–2.04)	-	2.25 (1.78–2.84)
Male	-	-	-	-	-	1.19 (1.03–1.39)
Affected Organs						
Brain/nerves	0.86 (0.82–0.91)	-	1.35 (1.19–1.55)	-	-	-
Skeleton/bone/muscle	0.86 (0.81–0.90)	-	1.22 (1.07–1.39)	-	0.62 (0.47–0.82)	-
Blood/bone marrow	0.86 (0.80–0.93)	-	-	-	-	-
Skin	0.87 (0.81–0.93)	-	-	-	-	-
Cancer	0.91 (0.86–0.96)	-	-	-	-	2.34 (1.88–2.90)
Infection	0.86 (0.81–0.91)	-	-	-	-	1.34 (1.04–1.73)
Eye/ear	-	1.12(1.02–1.22)	-	-	-	-
Lung	0.88 (0.85–0.92)	-	-	-	-	1.83 (1.50–2.24)
Liver	0.86 (0.81–0.91)	-	-	-	-	1.90 (1.45–2.49)
Comorbidities	-	-	-	-	-	-
Diabetes I/II	0.94 (0.91–0.97)		-	-	-	-
Stroke	0.90 (0.84–0.97)	-	-	-	-	-
Others	0.96 (0.93–0.99)	-	-	1.13 (1.01–1.27)	-	-
Setting characteristics						
Regions (ref. Eur A)						
American Region A	-	1.10 (1.02–1.17)	-	2.59 (2.06–3.26)	-	-
American Region B	0.92 (0.85–0.99)	-	-	-	-	1.82 (1.34–2.47)
Europe Region B/C	0.86 (0.79–0.93)	-	0.50 (0.30–0.83)	-	-	-
Japan	0.80 (0.68–0.93)	-	0.31 (0.23–0.43)	-	0.46 (0.31–0.70)	-
Specialty (ref. Internal medicine)	-	-	-	-	-	-
Cardiothoracic surgery	-	-	-	-	0.38 (0.15–0.95)	-
Ear Nose Throat (ENT)	-	-	-	-	0.22 (0.07–0.69)	-
General surgery	-	1.07 (1.00–1.14)	-	-	0.54 (0.39–0.76)	-
Geriatrics	0.67 (0.60–0.76)	-	-	-	0.67 (0.49–0.91)	-
Gynaecology	-	-	-	-	0.34 (0.13–0.94)	-
Long-term care	0.72 (0.53–0.99)	-	0.51 (0.29–0.91)	-	-	-
Orthopaedic surgery	-	-	-	-	0.47 (0.23–0.97)	-
Psychiatry	0.63 (0.45–0.87)	-	0.43 (0.21–0.88)	-	0.11 (0.02–0.70)	-
Nutrition-related characteristics						
Dietician available	-	-	0.77 (0.63–0.94)	-	-	-
Nutrition team available	0.95 (0.91–1.00)	-	-	-	-	-
Weight Δ in the last 3 months (reference idem)						
Lost weight	0.86 (0.83–0.88)	-	-	-	-	1.75 (1.40–2.18)
Unsure	0.87 (0.80–0.93)	-	-	1.47 (1.21–1.79)	-	4.50 (3.35–6.05)
Missing	0.83 (0.78–0.89)	-	-	1.32 (1.13–1.54)	-	2.88 (2.07–4.03)

Gray indicates determinant type of the variables that follow * statistically significant at *p* < 0.001 ** complete cases analysis using Cox proportional hazards model with competing risks extension. Definitions: American Region A = Canada, United States of America; American Region B = Argentina, Brazil, Chile, Columbia, Dominican Republic, Ecuador, El Salvador, Mexico, Panama, Paraguay, Uruguay; Asia Pacific = Bangladesh, China, India, Korea, Malaysia, Philippines, Singapore, Thailand; Eastern Mediterranean Region = Egypt, Iran, Kuwait, Oman, United Arab Emirates; Europe Region A = Austria, Belgium, Croatia, Cyprus, Czech Republic, Denmark, Finland, France, Germany, UK, Greece, Israel, Italy, Luxembourg, Netherlands, Norway, Slovenia, Spain, Sweden, Switzerland, Portugal; Europe Region B/C = Albania, Bosnia and Herzegovina, Bulgaria, Estonia, Georgia, Hungary, Latvia, Lithuania, Poland, Romania, Russian Federation, Serbia, Slovakia, Turkey, Ukraine; Japan = Japan; Oceania = Australia, New Zealand.

## Data Availability

Restrictions apply to the availability of these data. Data were obtained from nutritionDay^®^ and are available from Michael Hiesmayr with the permission of nutritionDay^®^. More information can be found at www.nutritionday.org [20].

## Supplementary Materials

The following are available online at https://www.mdpi.com/article/10.3390/nu13114111/s1, Table S1: Median length of stay by baseline variables adjusted for length bias, Table S2: Time to discharge country models 1–10: multivariable cause-specific Cox proportional hazards competing risks results for the outcome discharged, Table S3: Time to transfer country models 1–10: multivariable cause-specific Cox proportional hazards competing risks results for the outcome transferred, Table S4: Time to in-hospital death country models 1–10: multivariable cause-specific Cox proportional hazards competing risks results for the outcome died in hospital, Table S5: Time to discharge country models 11–20: multivariable cause-specific Cox proportional hazards competing risks results for the outcome discharged, Table S6: Time to transfer country models 11–20: multivariable cause-specific Cox proportional hazards competing risks results for the outcome transferred, Table S7: Time to in-hospital death country models 11–20: multivariable cause-specific Cox proportional hazards competing risks results for the outcome died in hospital, Table S8: Time to discharge country models 21–30: multivariable cause-specific Cox proportional hazards competing risks results for the outcome discharged, Table S9: Time to transfer country models 21–30: multivariable cause-specific Cox proportional hazards competing risks results for the outcome transferred, Table S10: Time to in-hospital death country models 21–30: multivariable cause-specific Cox proportional hazards competing risks results for the outcome died in hospital, Table S11: Baseline characteristics Figure S1: Global model: multivariable cause-specific Cox proportional hazards competing risks results.

## Appendix A

### Appendix A.1. Variables

The department bed occupancy was calculated using the number of patients in a department on nutritionDay divided by the number of beds available. The categories “low to middle”, “high capacity”, and “over capacity” were created using tertiles, in which “low to middle” capacity went from above 0 to 0.87, “high capacity” from above 0.87 to 0.99, and “overcapacity” from above 0.99 to 1.3 (the maximum). Patient characteristics used were age, sex, comorbidities, affected organs, and weight change in the last three months prior to hospitalization. The variable “year” was used to adjust for the varying annual participation cycle of different countries.

For the variable weight change in the last three months, it was possible to choose “weight gained”, “stayed the same (idem)”, “weight lost”, “I do not know”, or, if it was not filled out, then it was coded as “missing”. Nutrition team is on a hospital level while nutrition care person and dietician are on a department level. A nutrition care person is defined as any health professional of any grade who is responsible for or takes a leadership role in nutrition care in a specific department. A nutrition team is a multi-professional team including medical doctors, nurses, and pharmacists. Both were transformed into binary “yes” or “no” variables, with additional category of missing. Nutrition risk screening at admission refers to any method or screening tool used to detect whether or not malnutrition risk is present.

### Appendix A.2. Results

The percentage of complete cases was comparable throughout the event types: 80% complete case for discharged, 77% for transferred, 71% for died in hospital, and 78% for still in hospital at the 30-day follow-up (censored). Missingness appears to be random and not specifically associated with any variable (Table S1). Eleven percent of patients (10,218) were still in hospital at the 30-day follow-up after nutritionDay, 72% (*n* = 65,509) were discharged, 13% (*n* = 11,553) were transferred to another institution, and 3.5% (*n* = 3199) died in hospital (Figure 1). These proportions vary greatly between countries (Figure 2). Turkey had the highest proportion of discharged patients at 83%, while the Czech Republic had the lowest at 54%. The United States (US) had the lowest proportion of people dying in hospital with 1%, while Brazil had the highest at 8%. In terms of patients transferred, the Czech Republic had the highest proportion of transfer at 33%, while Thailand had the lowest at 3%. Japan had the highest number of patients still in hospital at follow-up at 25%, while the Netherlands had the lowest at 5%.
