# Supplementary material for: Predicting Hospital Length of Stay at Admission Using Global and Country-Specific Competing Risk Analysis of Structural, Patient, and Nutrition-Related Data from nutritionDay 2007–2015
Source: Nutrients. 2021 Nov 16;13(11):4111. doi: 10.3390/nu13114111 (PMC8624242; doi:10.3390/nu13114111)

Figure S1

Global model: multivariable cause-specific Cox proportional hazards competing risks results

	time to discharge (n=65,509) HR 99.9% CI	time to transfer (n=11,553) HR 99.9% CI	time to in-hospital death (n=3,199) HR 99.9% CI
AGE			
18-30	1.18 (1.12-1.24) *	0.80 (0.58-1.09)	0.31 (0.14-0.71) *
31-40	1.15 (1.09-1.21) *	0.82 (0.61-1.11)	0.49 (0.29-0.85) *
41-50	1.10 (1.05-1.15) *	0.84 (0.68-1.03)	0.78 (0.54-1.12)
51-60	1.06 (1.02-1.10) *	0.88 (0.74-1.04)	0.86 (0.65-1.15)
61-70	1.00	1.00	1.00
71-80	0.92 (0.89-0.96) *	1.25 (1.10-1.43) *	1.40 (1.10-1.77) *
81-120	0.78 (0.74-0.82) *	1.77 (1.54-2.04) *	2.25 (1.78-2.84) *
SEX			
Female	1.00	1.00	1.00
Male	1.00 (0.97-1.02)	0.95 (0.88-1.04)	1.19 (1.03-1.39) *
AFFECTED ORGANS			
Brain/nerves	0.86 (0.82-0.91) *	1.35 (1.19-1.55) *	1.21 (0.95-1.54)
Skeleton/bone/muscle	0.86 (0.81-0.90) *	1.22 (1.07-1.39) *	0.62 (0.47-0.82) *
Blood/bone marrow	0.86 (0.80-0.93) *	0.99 (0.80-1.23)	1.04 (0.76-1.43)
Skin	0.87 (0.81-0.93) *	0.98 (0.78-1.22)	1.06 (0.68-1.63)
Ischaemia	0.89 (0.78-1.00)	1.11 (0.78-1.58)	1.05 (0.58-1.89)
Cancer	0.91 (0.86-0.96) *	0.87 (0.70-1.08)	2.34 (1.88-2.90) *
Infection	0.86 (0.81-0.91) *	0.96 (0.83-1.11)	1.34 (1.04-1.73) *
Others	1.03 (0.93-1.13)	0.99 (0.78-1.26)	1.23 (0.86-1.75)
Eye/ear	1.12 (1.02-1.22) *	0.83 (0.61-1.13)	0.75 (0.34-1.67)
Nose/throat	1.07 (0.98-1.17)	1.03 (0.75-1.40)	0.75 (0.47-1.18)
Heart/circulation	1.04 (1.00-1.09)	1.02 (0.89-1.16)	1.00 (0.81-1.23)
Lung	0.88 (0.85-0.92) *	0.94 (0.83-1.07)	1.83 (1.50-2.24) *
Liver	0.86 (0.81-0.91) *	0.94 (0.77-1.16)	1.90 (1.45-2.49) *
Gastrointestinal	0.98 (0.94-1.02)	0.96 (0.83-1.10)	0.90 (0.73-1.11)
Kidney/urinary/genital	0.96 (0.91-1.01)	1.00 (0.84-1.18)	1.01 (0.82-1.26)
Endocrine system	1.00 (0.95-1.05)	0.92 (0.78-1.09)	0.82 (0.59-1.15)
COMORBIDITIES			
Diabetes I/II	0.94 (0.91-0.97) *	0.99 (0.89-1.09)	1.00 (0.82-1.22)
Stroke	0.90 (0.84-0.97) *	1.15 (1.00-1.32)	1.09 (0.81-1.46)
COPD	0.96 (0.91-1.01)	1.02 (0.88-1.19)	0.93 (0.72-1.21)
Myocardial infarction	0.96 (0.89-1.03)	1.06 (0.88-1.29)	1.19 (0.84-1.70)
Cardiac insufficiency	0.96 (0.92-1.00)	1.02 (0.91-1.16)	1.18 (0.95-1.47)
Others	0.96 (0.93-0.99) *	1.13 (1.01-1.27) *	1.16 (0.98-1.38)
REGIONS			
AMR A	1.10 (1.02-1.17) *	2.59 (2.06-3.26) *	0.66 (0.41-1.04)
AMR B	0.92 (0.85-0.99) *	0.68 (0.37-1.26)	1.82 (1.34-2.47) *
Asia Pacific	1.06 (0.94-1.20)	0.78 (0.49-1.26)	0.76 (0.46-1.25)
EMR	0.94 (0.83-1.07)	0.99 (0.36-2.71)	1.49 (0.65-3.41)
EUR A	1.00	1.00	1.00
EUR B/C	0.86 (0.79-0.93) *	0.50 (0.30-0.83) *	0.86 (0.55-1.35)
Japan	0.80 (0.68-0.93) *	0.31 (0.23-0.43) *	0.46 (0.31-0.70) *
Ozeania	1.08 (0.89-1.31)	1.00 (0.61-1.64)	0.56 (0.19-1.69)
YEAR			
Year 1	1.00	1.00	1.00
Year 2	0.98 (0.89-1.09)	1.24 (0.83-1.86)	1.24 (0.79-1.97)
Year 3	1.06 (0.96-1.18)	1.08 (0.70-1.67)	1.04 (0.66-1.64)
Year 4	1.04 (0.94-1.14)	0.99 (0.65-1.52)	1.09 (0.70-1.68)
Year 5	1.10 (0.99-1.22)	1.25 (0.82-1.91)	1.30 (0.81-2.08)
Year 6	1.14 (1.03-1.27) *	1.14 (0.77-1.70)	1.28 (0.82-2.00)
Year 7	1.15 (1.03-1.27) *	1.26 (0.87-1.83)	1.06 (0.66-1.68)
Year 8	1.20 (1.08-1.34) *	1.37 (0.95-1.98)	1.06 (0.66-1.71)
Year 9	1.07 (0.97-1.19)	1.74 (1.20-2.51) *	1.14 (0.73-1.80)
SPECIALTY			
Cardiology	1.06 (0.96-1.16)	0.91 (0.66-1.26)	1.01 (0.61-1.68)
Cardiothoracic surgery	0.84 (0.67-1.05)	1.42 (0.85-2.36)	0.38 (0.15-0.95) *
Ear Nose Throat (ENT)	0.97 (0.84-1.11)	0.58 (0.25-1.38)	0.22 (0.07-0.69) *
Gastroenterology	1.03 (0.94-1.13)	1.01 (0.69-1.47)	0.96 (0.67-1.38)
General surgery	1.07 (1.00-1.14) *	0.78 (0.56-1.07)	0.54 (0.39-0.76) *
Geriatrics	0.67 (0.60-0.76) *	0.89 (0.70-1.11)	0.67 (0.49-0.91) *
Gynaecology	1.09 (0.94-1.26)	1.05 (0.29-3.87)	0.34 (0.13-0.94) *
Infections	0.97 (0.83-1.14)	0.95 (0.58-1.57)	0.72 (0.34-1.54)
Internal medicine	1.00	1.00	1.00
Long-term-care	0.72 (0.53-0.99) *	0.51 (0.29-0.91) *	0.38 (0.14-1.02)
Nephrology	0.98 (0.73-1.32)	1.26 (0.75-2.13)	0.60 (0.26-1.40)
Neurology	0.98 (0.87-1.11)	0.99 (0.70-1.40)	0.61 (0.33-1.12)
Neurosurgery	0.93 (0.76-1.14)	1.42 (0.83-2.42)	0.33 (0.05-2.11)
Oncology	1.01 (0.92-1.10)	0.71 (0.43-1.16)	1.16 (0.82-1.62)
Orthopaedic surgery	0.93 (0.83-1.03)	1.20 (0.86-1.67)	0.47 (0.23-0.97) *
Others	1.03 (0.94-1.13)	0.81 (0.58-1.12)	0.70 (0.48-1.01)
Pediatrics	1.24 (0.60-2.55)	0.75 (0.12-4.94)	1.36 (0.40-4.68)
Psychiatry	0.63 (0.45-0.87) *	0.43 (0.21-0.88) *	0.11 (0.02-0.70) *
Trauma	1.06 (0.93-1.21)	1.16 (0.68-1.96)	0.50 (0.13-1.86)
BED OCCUPANCY			
High occupancy	0.98 (0.93-1.03)	0.90 (0.75-1.10)	0.92 (0.73-1.17)
Low to middle occupancy	1.00	1.00	1.00
Over occupied	1.00 (0.95-1.05)	0.88 (0.73-1.06)	0.91 (0.72-1.15)
DIETICIAN AVAILABILITY			
Available	1.05 (1.00-1.11)	0.77 (0.63-0.94) *	1.08 (0.85-1.35)
Missing	1.04 (0.97-1.12)	0.89 (0.67-1.19)	1.03 (0.72-1.49)
None available	1.00	1.00	1.00
NUTRITION TEAM			
No	1.00	1.00	1.00
Yes	0.95 (0.91-1.00) *	0.92 (0.78-1.07)	1.04 (0.82-1.32)
NUTRITIONAL CARE PERSON			
No	1.00	1.00	1.00
Yes	1.02 (0.96-1.07)	1.02 (0.84-1.24)	0.83 (0.66-1.05)
SCREENING AT ADMISSION			
Missing	1.00 (0.80-1.25)	0.73 (0.19-2.77)	1.82 (0.39-8.45)
Not Screened at Admission	1.00	1.00	1.00
Screened at Admission	1.01 (0.96-1.06)	1.18 (0.97-1.43)	1.03 (0.83-1.27)
WEIGHT CHANGE LAST 3 MONTHS			
Gained Weight	1.00 (0.96-1.05)	1.04 (0.87-1.25)	0.89 (0.56-1.42)
Idem	1.00	1.00	1.00
Lost Weight	0.86 (0.83-0.88) *	1.07 (0.97-1.19)	1.75 (1.40-2.18) *
Missing	0.87 (0.80-0.93) *	1.47 (1.21-1.79) *	4.50 (3.35-6.05) *
Unsure	0.83 (0.78-0.89) *	1.32 (1.13-1.54) *	2.88 (2.07-4.03) *

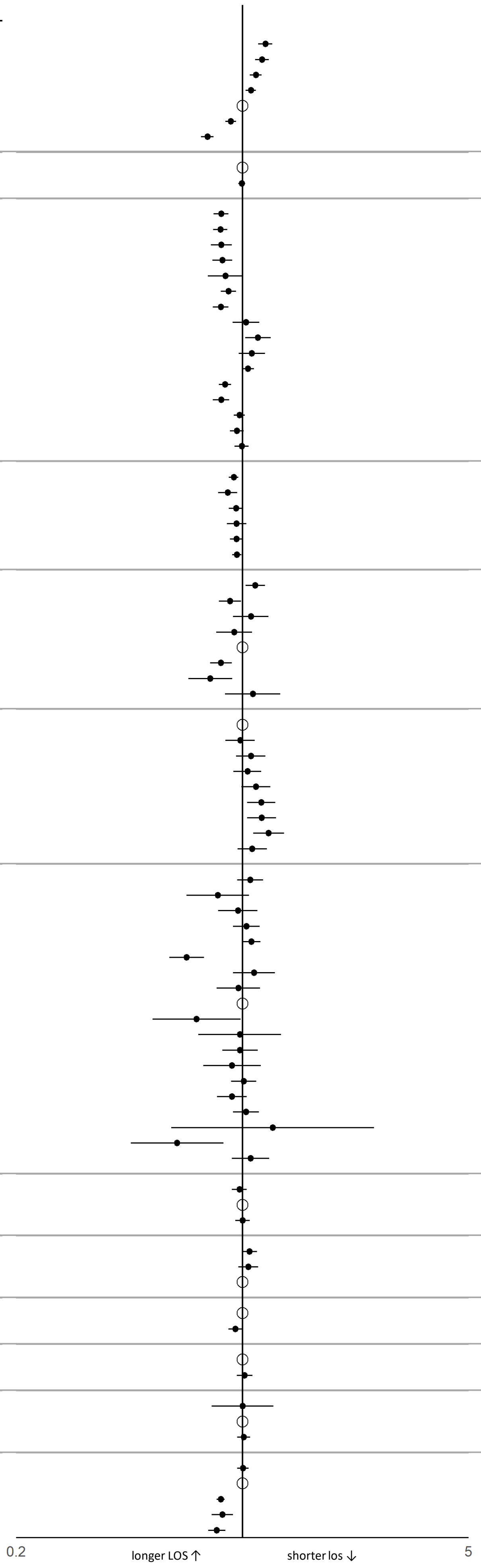

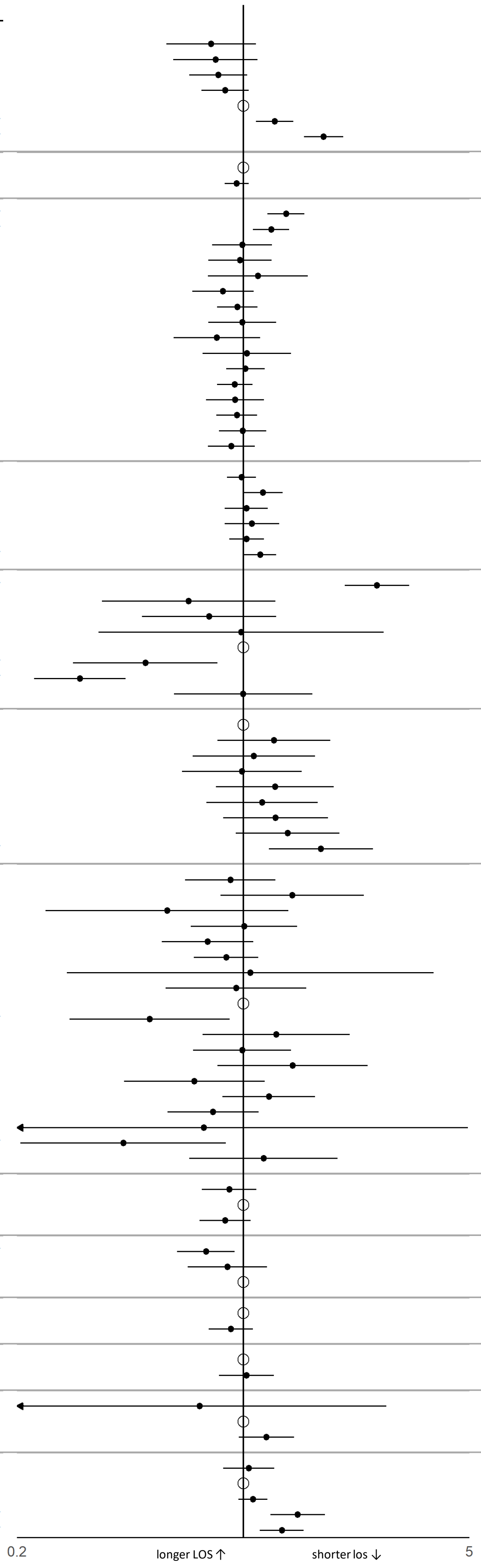

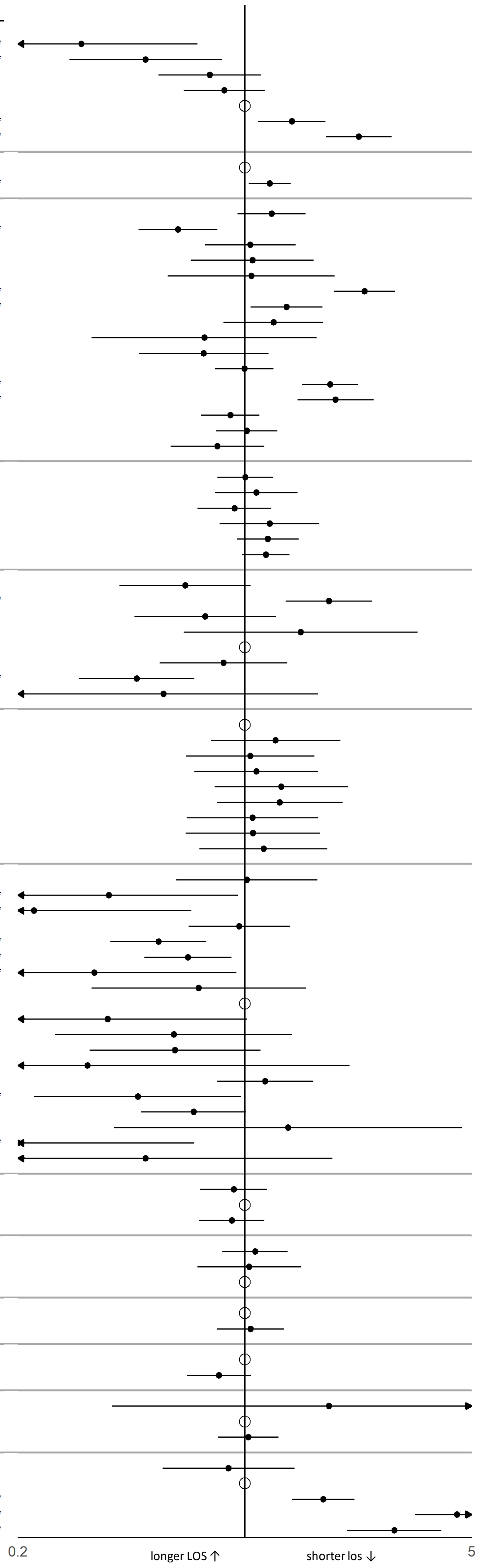

Supplement: Supplementary file 1 [file nutrients-13-04111-s001.zip › Supplementary Figure S1 2021-11-02.pdf]
